# Lyn, a tyrosine kinase closely linked to the differentiation status of primary acute myeloid leukemia blasts, associates with negative regulation of all-*trans* retinoic acid (ATRA) and dihydroxyvitamin D3 (VD3)-induced HL-60 cells differentiation

**DOI:** 10.1186/s12935-016-0314-5

**Published:** 2016-05-13

**Authors:** Noriyoshi Iriyama, Bo Yuan, Yoshihiro Hatta, Norio Takagi, Masami Takei

**Affiliations:** Division of Hematology and Rheumatology, Department of Medicine, Nihon University School of Medicine, Itabashi Hospital, 30-1 Oyaguchi Kamicho, Itabashi-ku, Tokyo, Japan; Department of Applied Biochemistry, School of Pharmacy, Tokyo University of Pharmacy and Life Sciences, 1432-1 Horinouchi, Hachioji, Tokyo 192-0392 Japan

**Keywords:** Acute myeloid leukemia, Lyn, dasatinib, All-*trans* retinoic acid, Dihydroxyvitamin D3, c-Myc

## Abstract

**Background:**

Lyn, an import member of Src family kinases (SFKs), is supposed to be implicated in acute myeloid leukemia (AML) pathogenesis and development by participation in AML differentiation, yet the details still remain incompletely understood. The expression status of Lyn and its correlation with multiple clinical parameters including cell differentiation degree, different cytogenetic risk classification, and the activity of myeloperoxidase (MPO) were thus investigated. To address the mechanisms underlying the involvement of Lyn in differentiation induction, the effects of dasatinib, an inhibitor for SFKs including Lyn, on the alterations of all-*trans* retinoic acid (ATRA)- or dihydroxyvitamin D3 (VD3)-induced differentiation, and c-Myc protein expression were investigated.

**Methods:**

Primary AML blasts were obtained from 31 newly diagnosed AML patients with different French-American-British (FAB) subtypes. The expression of phosphorylated and total Lyn, c-Myc, and CD11b, CD11c and CD15 was analyzed by flow cytometry. The activation of Akt and Erk known to be involved in the regulation of c-Myc expression was investigated using western blotting.

**Results:**

Significant higher expression levels of total Lyn were observed in AML patients with favorable cytogenetics, higher MPO activity and FAB M2 subtype. A clear positive correlation between the expression levels of Lyn and differentiation status of primary AML blasts was observed. Dasatinib inhibited the expression of phosphorylated Lyn, and further enhanced the differentiation-inducing activity of ATRA and VD3 in HL-60 cells. Augmented downregulation of c-Myc protein expression was observed in the combination treatment with ATRA, VD3 and dasatinib compared to treatment with each reagent alone in HL-60 cells. The suppression of the activation of Akt and Erk was also observed concomitantly.

**Conclusions:**

The expression level of total Lyn is closely linked to the differentiation status of AML blasts. The enhancement of differentiation-inducing activity of ATRA/VD3 by dasatinib suggested that Lyn was associated in the negative regulation of ATRA/VD3-induced HL-60 cells differentiation. The enhancement probably was attributed to the downregulation of c-Myc implicated with the suppression of the activation of Akt and Erk. These results provide novel insights into a possible combinational therapeutic approach by targeting Lyn for AML patients, and offer new possibilities for the combination therapy with VD3 and dasatinib.

## Background

Acute myeloid leukemia (AML) is one of the major hematologic neoplasms, and is characterized by defective differentiation and excessive accumulation of proliferative progenitor cells in the bone marrow and blood. AML has several subtypes and its treatment and prognosis varies among subtypes. Although conventional and/or advanced treatment strategy employing all-*trans* retinoic acid (ATRA) in combination with other drugs including arsenic trioxide has dramatically improved the prognosis for acute promyelocytic leukemia (APL) [1–3], unfavorable outcomes due to the relapse and drug resistance are still a serious concern in other subtype of AML with five-year disease-free survival rates of less than 50 % [4–6]. Because of the lack of effective molecular-targeting agents, the treatment strategy for non-APL AML is limited to chemotherapy with or without hematopoietic stem cell transplantation to date.

Src family kinases (SFKs) are a unique group of enzymes that have critical roles in cell proliferation, survival, differentiation, adhesion and migration in leukemias and solid tumors, as well as in embryonic stem cell development [7–9]. Pharmacologic inhibition of SFKs by their inhibitors, such as PP2 or dasatinib, inhibits the growth of human leukemia cell lines and primary AML blast cells [10, 11]. Lyn, an important SFK family member, expressed in an active form in AML cells and is supposed to play a critical role in AML differentiation and known to be responsible for imatinib-resistant leukemias [10, 12, 13]. Especially, dasatinib has proven successful in the treatment of imatinib-resistant leukemias in which the effects of dasatinib are likely attributed to the inhibition of Lyn [12, 14–16]. Although these previous findings strongly implicate Lyn in AML pathogenesis or disease development, the detailed systematic analysis of the expression status of Lyn in different subtypes of AML has not yet been preformed.

HL-60 cells are known as a good model for leukemogenesis research and differentiation study in vitro [17, 18]. Interestingly, Lyn has been demonstrated to be the predominant activated SFK in HL-60 cells, although other SFK members such as Lck, Fyn, Hck also expressed in the cells [12]. However, the detailed investigation focused on the involvement of Lyn in the induction of differentiation in HL-60 cells by different stimulants has not yet been sufficiently conducted.

The c-Myc transcription factor is a potent regulator of a variety of cell activities such as proliferation and differentiation [19]. Deregulated c-Myc expression is common in the highly proliferative leukemias and lymphomas which are blocked at an earlier stage of differentiation [20–22]. In fact, repression of c-Myc has been reported to be required for terminal differentiation of malignant hematopoietic cells including HL-60 cells induced by different stimulants such as ATRA and dihydroxyvitamin D3 (VD3) [23–26]. However, details of the correlation between c-Myc and Lyn still remain incompletely understood in view of their roles in differentiation induction.

In this study, based on our hypothesis that Lyn expression may differ in individual patients and could be related with some clinical parameters due to the heterogeneity of AML, the expression status of total and phosphorylated Lyn in primary AML cells from patients with different subtypes of AML were first investigated. The correlation between Lyn expression status and multiple clinical parameters including cell differentiation degree, different cytogenetic risk classification, and the activity of myeloperoxidase (MPO) were further evaluated. In order to address the mechanisms underlying the involvement of Lyn in differentiation induction, the effects of dasatinib, an inhibitor for SFKs including Lyn, on the alterations of ATRA- or VD3-induced differentiation, the expression of c-Myc in HL-60 cells were further investigated. Since Akt and Erk have been demonstrated to be involved in complex signaling pathways responsible for the regulation of c-Myc expression [19, 27–29], the activation of Akt and Erk was further investigated.

## Methods

### Cell line and materials

HL-60, a human non-APL AML cell line, was obtained from the Health Science Research Resources Bank (Tokyo, Japan). ATRA and VD3 were purchased from Sigma (St. Louis, MO, USA) and dissolved in ethanol to obtain a final concentration of 1 mM and 100 μM, respectively, and stored at −20 °C in the dark. Dasatinib was purchased from BioVision (Mountain View, CA, USA) and dissolved in dimethyl sulfoxide (DMSO) at a concentration of 1 mM. Phycoerythrin (PE)-conjugated mouse anti-human CD11b IgG, fluorescein isothiocyanate (FITC)-labeled mouse anti-human CD11c IgG, and the PE and FITC-conjugated isotype control IgG were obtained from Becton–Dickinson (San Jose, CA, USA). FITC-labeled polyclonal antibodies for total and phosphorylated Lyn (Y396) were purchased from Bioss (Woburn, MA, USA). Rabbit anti-human c-Myc polyclonal antibody and its negative control (non-binding rabbit IgG) were purchased from GeneTex (Irvine, CA, USA). FITC-labeled goat anti-rabbit polyclonal IgG antibody (secondary antibody) was purchased from Jackson ImmunoResearch (West Grove, PA, USA).

### Preparation of primary AML blasts

Primary AML blasts were obtained from 31 newly diagnosed AML patients with different French-American-British (FAB) subtypes, except for FAB M3 (APL). Prior to clinical therapy, mononuclear cell fractions were isolated from freshly collected bone marrow aspirates or peripheral blood using Lymphoprep™ (Cosmo Bio Co., Ltd., Tokyo, Japan), and the cell fractions were then cryopreserved in liquid nitrogen until use. Prior to conducting the experiments, aliquots of the AML cells were rapidly thawed at 37 °C and washed with PBS. Cells were then stained with Wright-Giemsa solution to confirm blastoid cell content and more than 90 % of blastoid cell fraction was ensured in all samples. The study was approved by the Internal Review Committee of Nihon University Itabashi Hospital.

### Cell culture and treatment

HL-60 cells were maintained in RPMI 1640 medium (Sigma) supplemented with 10 % heat-inactivated fetal bovine serum (FBS) (Gibco BRL, Grand Island, NY, USA), 100 U/ml penicillin, and 100 μg/ml streptomycin (Gibco BRL) at 37 °C in a humidified atmosphere (5 % CO_2_ in air). Cells were seeded at a density of 1 × 10^5^ cells/ml and treated with 1 μM ATRA, 100 nM VD3, 300 nM dasatinib, alone or in combination, for the indicated time. Dose titration study revealed that less than or equal to 1 μM of dasatinib had no apoptotic effect on HL-60 cells (data not shown). Hence, we applied 300 nM of concentration as shown previously [14].

### Differentiation analysis

Differentiation induction was confirmed by morphology and expression of surface markers CD11b and CD11c. For morphological assessment, cytospin preparations of treated cells stained with Wright-Giemsa were evaluated by light microscopy as previously described [30, 31]. Myeloid maturation with cell surface marker was analyzed by flow cytometry (FACSCalibur, Becton–Dickinson, Franklin Lakes, NJ), as described previously [31, 32]. In brief, approximately 1 × 10^6^ cells were washed with phosphate-buffered saline (PBS) containing 2.5 % FBS and 0.5 % NaN_3_ (PBSF) and stained with anti-CD11b and CD11c antibodies for 30 min at 4 °C in the dark. Cells were then washed three times with PBSF and analyzed by flow cytometry with a minimum acquisition of 10,000 events for HL-60 and 5000 events for primary AML cells prepared from patients, respectively. Non-binding mouse PE and FITC-labeled IgG was used as control. Assessment of the expression of CD15 by flow cytometry was entrusted to Bio Medical Laboratories, Inc. (BML, Inc., Tokyo, Japan).

### Detection of the expression of total, phosphorylated Lyn and c-Myc

Cells were harvested and washed once with PBS, and further fixed with 4 % formaldehyde/PBS at 37 °C for 10 min. After permeabilization with 90 % ice-cold methanol at −20 °C over 2 h, cells were washed once with PBSF and stained with anti-total, phosphorylated Lyn, and c-Myc antibody, respectively. FITC-labeled anti-rabbit secondary antibody was used for the detection of c-Myc. The expression levels of total and phosphorylated Lyn in primary AML cells were analyzed by flow cytometer (Cyto ACE-150, Jasco, Tokyo, Japan), and the results were shown as mean fluorescence intensity (MFI) with linear scaling and calculated by subtracting the mean fluorescence of the unstained cells from that of the stained cells, as described previously [30, 32–34]. Similarly, the expression levels of phosphorylated Lyn in HL-60 cells were also analyzed by flow cytometry (FACSCalibur) and were shown as MFI. The expression levels of c-Myc were evaluated using flow cytometer (Cyto ACE-150) and were shown as positive rates.

### Western blot

Protein samples were separated on an SDS-PAGE, followed by transferring to a nitrocellulose membrane as described previously [30]. Protein bands were detected using the following primary antibodies: mouse anti-human β-actin (1:5000 dilution, Sigma-Aldrich); rabbit anti-human phospho-p44/42 MAPK (Erk1/2) (Thr202/Tyr204) (1:2000 dilution) and p44/42 MAPK (Erk1/2) (1:1000 dilution); rabbit anti-human phospho-Akt (Ser473) (1:2000 dilution) and Akt (1:1000 dilution) (Cell Signaling Technology, MA, USA). Blotted protein bands were detected with respective horseradish peroxidase-conjugated secondary antibody and an enhanced chemiluminescence (ECL) western blot analysis system (Amersham Pharmacia Biotech, Buckinghamshire, UK).

### Statistical analysis

Experiments were independently repeated more than three times and results are shown as mean ± standard deviation (SD). A two-tailed paired *t* test was used to assess the difference between two groups. The correlation in two factors was evaluated with Pearson product-moment correlation coefficient. A *p* value less than 0.05 was considered to be significant.

## Results

### Correlation between Lyn expression level and clinical laboratory parameters

**Fig. 1:**
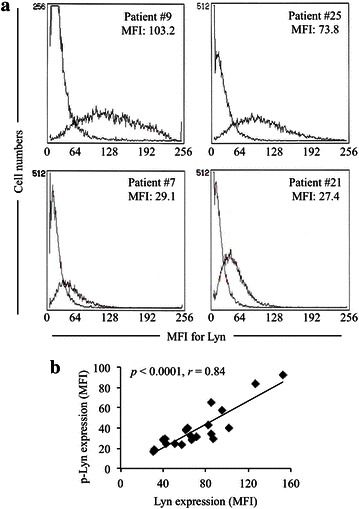
Representative flow cytometry histograms of Lyn expression and a positive correlation between the expression levels of total and phosphorylated Lyn in primary AML blast cells from patients. The expression levels of Lyn in primary AML cells were analyzed and presented as histograms plotted by flow cytometry as described in “Methods” section. **a** Histograms show the high (patients #9 and #25) and low (patients #7 and #21) Lyn expression level in respective primary AML blast cells of individual patients. **b** A strong correlation between the expression levels of total and phosphorylated Lyn (p-Lyn). *MFI* mean fluorescence intensity

**Fig. 2:**
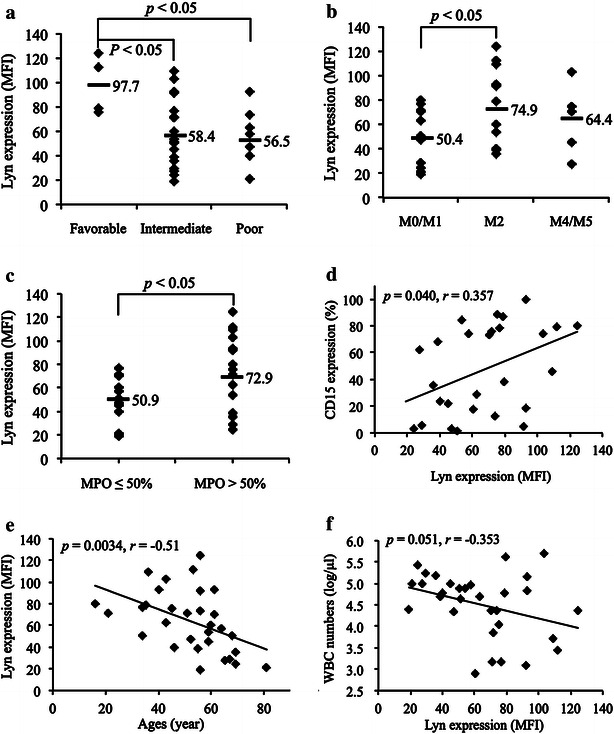
Correlation between the expression level of Lyn and clinical laboratory parameters. The expression levels of Lyn in primary AML cells obtained from 31 patients with different subtypes of AML were analyzed with flow cytometry. Multiple clinical parameters of individual patients were collected in Nihon University Itabashi Hospital, and the assessment of the expression level of CD15 was entrusted to Bio Medical Laboratories, Inc. The correlation between the expression levels of Lyn and different cytogenetic risk groups (**a**), different AML subtypes (**b**), MPO activity (**c**), the expression level of CD15 (**d**), patients’ age (**e**) and WBC numbers (**f**) were shown respectively

Lyn has been demonstrated to be the major SFK family member expressed in an active form in AML cells [10, 12], the expression levels of Lyn in primary AML cells from 31 patients with different subtypes of AML were thus investigated and summarized in Table 1. Multiple clinical parameters of individual patients enrolled into the study were also shown in Table 1. Flow cytometry data for representative samples with high or low Lyn expression level were shown in Fig. 1a. The expression levels of phosphorylated Lyn in 20 samples of the 31 primary AML blasts were further evaluated. A strong positive correlation between the expression levels of total and phosphorylated Lyn was observed (Fig. 1b), suggesting its biological and clinical significance in AML pathogenesis and development. Intriguingly, significant higher expression levels of Lyn were observed in AML patients with favorable cytogenetics compared to intermediate or poor cytogenetic risk group according to MRC10 criteria [35] (Fig. 2a; Table 1). Much higher expression levels of Lyn were observed in patients with FAB M2 subtype compared to all other FAB subtypes, and a significant difference in its expression level was further confirmed between patients with FAB M2 and FAB M0/M1 (Fig. 2b; Table 1). Significant higher expression levels of Lyn were also observed in patients with higher myeloperoxidase (MPO) activity (more than 50 % MPO-positive blasts), known to be closely associated with cell differentiation status (Fig. 2c; Table 1). Consistent with this, a positive correlation was observed between the expression levels of Lyn and CD15, a major myeloid maturation marker (Fig. 2d). Interestingly, there was a strong inverse correlation between the expression levels of Lyn and patients’ age (Fig. 2e), whereas, no correlation was observed between its expression and the white blood cell (WBC) numbers of patients (Fig. 2f).

**Table 1:** Multiple clinical parameters of 31 AML patients and Lyn expression status of primary AML blasts

Patient number	Age	Sex	Diagnosis	Karyotype	WBC numbers (/μl)	MPO positivity (%)	Lyn intensity (MFI)
1	45	M	M4E	inv (16) (p13;q22)	10,900	88	75.3
2	56	F	M2	t (8;21) (q22;q22)	23,700	100	124.5
3	53	F	M2	t (8;21) (q22;q22), -X	2800	100	112.1
4	35	M	M2	t (8;21) (q22;q22), del(11) (p13), +15	59,300	ND	78.8
5	59	M	M2	21 trisomy	78,200	100	53.9
6	69	F	M1	NK	264,700	100	24.2
7	67	M	M1	NK	168,800	100	29.1
8	34	M	M0	NK	1500	2	76.9
9	43	M	M4	NK	512,800	91	103.2
10	21	M	M1	t (X;5) (q28;q31)	6900	90	71.9
11	60	M	M2	NK	800	6.5	60.3
12	36	M	M2	NK	5300	97	109.3
13	56	M	M2	NK	1200	94	91.8
14	40	F	M2	NK	69,100	99	92.9
15	34	M	M1	NK	43,400	20	51.1
16	59	M	M4	NK	99,200	39.5	45
17	69	F	M2	NK	149,000	98.5	35.8
18	68	M	MDS/AML	add (11) (q23)	78,700	23.5	50.2
19	55	M	M2	NK	49,400	90	38.8
20	51	F	M5a	t (9;11) (p22;q23), +8	1500	0	71
21	65	M	M4	NK	96,400	ND	27.4
22	56	M	M1	NK	25,300	17.2	19.1
23	61	M	M2	Complex	140,300	92	92.8
24	46	M	M2	Complex	60,600	31	40.2
25	56	M	MDS/AML	Complex	23,000	ND	73.8
26	43	F	M1	Complex	50,600	100	63
27	81	F	M0	t (9;22) (q34;q11), add(7) (p13), −22	95,300	1	21.1
28	52	M	M0	Complex	22,300	1.5	47.2
29	64	M	MDS/AML	Complex	93,200	9.5	57.6
30	16	M	M1	ND	403,300	93.5	79.7
31	61	M	M1	ND	23,100	9	70.6

Primary AML blasts were obtained from 31 newly diagnosed AML patients with different FAB subtypes, except for FAB M3 (APL). The Lyn expression status of primary AML blasts from individual patients was analyzed by flow cytometer, and shown as mean fluorescence intensity (MFI). Multiple clinical parameters of individual patients were collected in Nihon University Itabashi Hospital

*WBC* white blood cells, *MPO* myeloperoxidase, *NK* normal karyotype, *MDS/AML* myelodysplastic syndrome overt acute myeloid leukemia, *ND* not detected

### Inhibition of phosphorylation of Lyn and enhancement of differentiation-inducing activity of both ATRA and VD3 by dasatinib in HL-60 cells

**Fig. 3:**
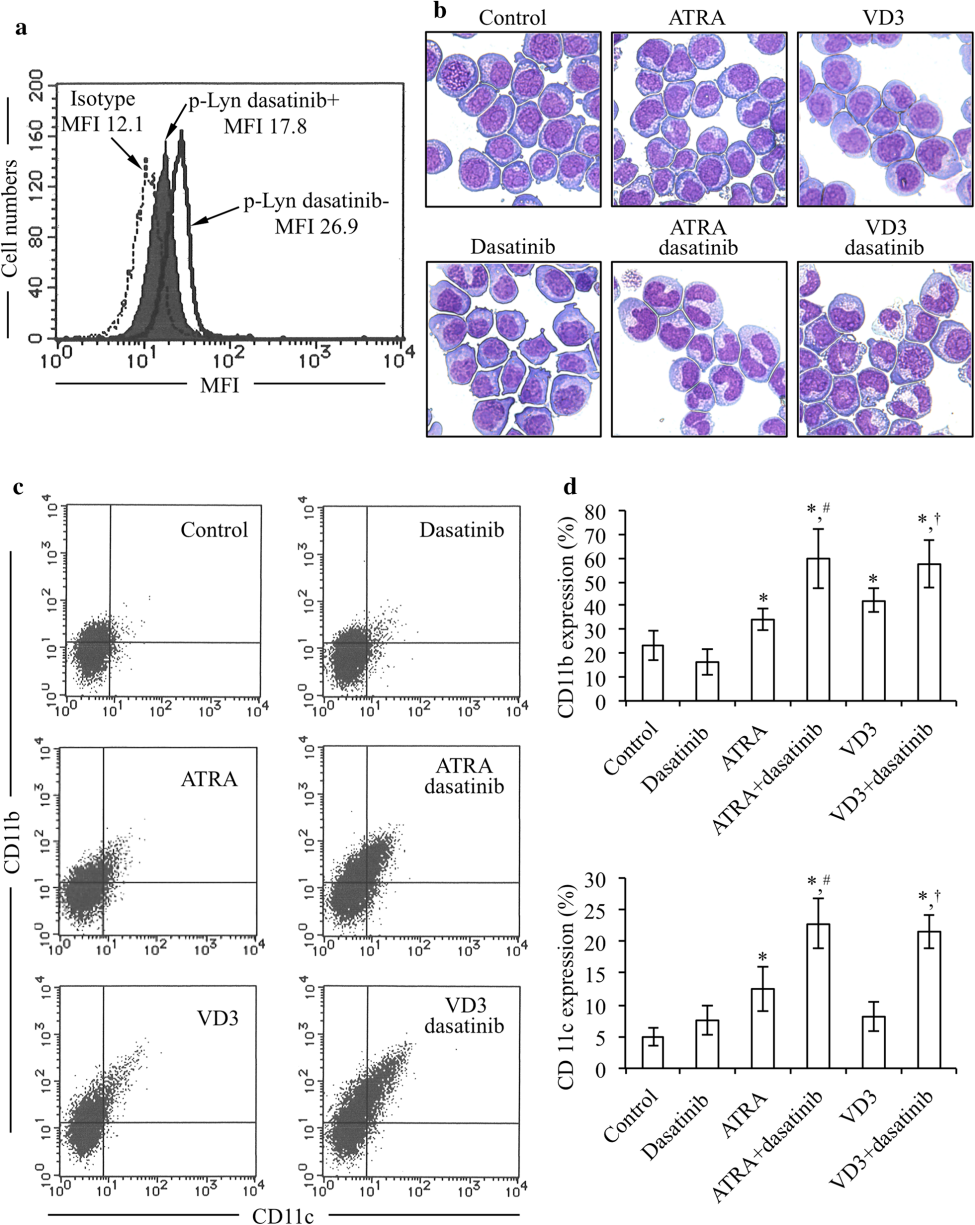
Inhibition of phosphorylation of Lyn and enhancement of differentiation-inducing activity of both ATRA and VD3 by dasatinib in HL-60 cells. **a** After treatment with 300 nM dasatinib for 30 min, the expression levels of phosphorylation of Lyn were evaluated by flow cytometry as described in “Methods” section. **b**–**d** After treatment with 1 μM ATRA or 100 nM VD3 in the presence or absence of 300 nM dasatinib for 72 h, morphological changes and the expression profiles of CD11b and CD11c were evaluated by Wright-Giemsa staining, and flow cytometry, respectively, as described in “Methods” section. **b** Representative photomicrographs are shown from three independent experiments. **c** Flow cytometry profiles of CD11b and CD11c. **d** Percent of CD11b and CD11c positive cells were calculated, respectively, based on flow cytometry profiles shown in (**c**). Experiments were independently repeated at least three times and results are shown as mean ± SD). p-Lyn dasatinib+, the expression level of phosphorylated Lyn in the presence of dasatinib; p-Lyn dasatinib−, the expression level of phosphorylated Lyn in the presence of dasatinib. *p < 0.05 vs. control; ^#^ *p* < 0.05 vs. ATRA; ^†^ *p* < 0.05 vs. VD3

Lyn plays a critical role in AML differentiation, and is the predominant activated SFK in AML cells such as HL-60 [10, 12]. Flow cytometric analysis was thus first performed to confirm the expression status of phosphorylated Lyn and clarify the effect of dasatinib, an inhibitor for SFKs including Lyn, on its expression level in HL-60 cells after treatment with 300 nM dasatinib for 30 min. Consistent with previous reports [10, 12], the expression of phosphorylated Lyn was confirmed, and an apparent inhibitory effect of dasatinib on its expression was observed (Fig. 3a). To determine the effects of dasatinib on ATRA- or VD3-induced differentiation, HL-60 cells were treated with 1 μM ATRA or 100 nM VD3 in the presence of absence of 300 nM dasatinib for 72 h, followed by morphological analysis and flow cytometric analysis of the myeloid differentiation markers CD11b and CD11c (Fig. 3b–d). HL-60 cells treated with ATRA or VD3 alone underwent apparent differentiation-associated changes with condensation and lobulation of nuclei (Fig. 3b), accompanied by a significant increase in the expression levels of CD11b and/or CD11c (Fig. 3c, d). Furthermore, the number of cells containing multi-lobulated nuclei prominently increased when cells were treated with ATRA or VD3 in combination with dasatinib compared to when treated with ATRA or VD3 alone (Fig. 3b). The enhancement in differentiation-inducing activity due to the combination treatment was further confirmed by a significant increase in the expression levels of CD11b and CD11c (Fig. 3c, d). Moreover, in comparison to control groups, no morphological change and alteration in the expression levels of the two differentiation markers was observed when treated by dasatinib alone (Fig. 3b–d).

### Potentiation of ATRA- or VD3-induced c-Myc downregulation by dasatinib in HL-60 cells

**Fig. 4:**
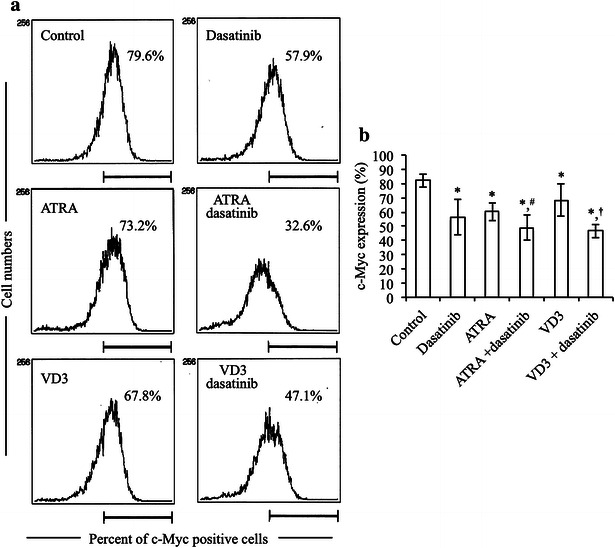
Potentiation of ATRA- or VD3-induced c-Myc downregulation by dasatinib in HL-60 cells. After treatment with 1 μM ATRA, 100 nM VD3, alone or in combination with 300 nM dasatinib, for 72 h, the alteration of c-Myc expression in HL-60 cells was examined by intracytoplasmic staining using flow cytometry as described in “Methods” section. The relative expression level of c-Myc is shown as the mean fluorescence intensity (MFI). **a** Representative histogram rofiles are shown. **b** The relative expression level of c-Myc was calculated based on flow cytometry profiles shown in (**a**). Six independent experiments were carried out and results are shown as mean ± SD. *p < 0.05 vs. control; ^#^ *p* < 0.05 vs. ATRA; ^†^ *p* < 0.05 vs. VD3

Since deregulated c-Myc expression is common in the highly proliferative leukemias and lymphomas which are blocked at an earlier stage of differentiation [20–22], the alteration of c-Myc expression in HL-60 cells was examined by intracytoplasmic staining using flow cytometry after treatment with 1 μM ATRA and 100 nM VD3, each alone or in combination with 300 nM dasatinib for 72 h. As shown in Fig. 4, ATRA, VD3 and dasatinib alone induced slightly but significantly downregulation of c-Myc expression. Furthermore, dasatinib significantly potentiated the capacity of both ATRA and VD3 to downregulate the expression level of c-Myc.

### Expression profiles of phosphorylated and total Akt and Erk in HL-60 cells treated with ATRA and VD3, each alone or in combination with dasatinib

**Fig. 5:**
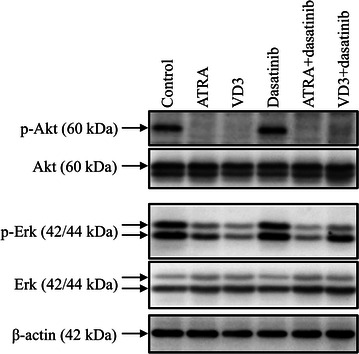
Expression profiles of phosphorylated and total Akt and Erk in HL-60 cells treated with ATRA and VD3, each alone or in combination with dasatinib. After treatment with 1 μM ATRA and 100 nM VD3, each alone or in combination with 300 nM dasatinib for 24 h, the alterations of the expression level of phosphorylated and total Akt and Erk proteins were evaluated by western blotting method as described in “Methods” section

After treatment with 1 μM ATRA and 100 nM VD3, each alone or in combination with 300 nM dasatinib for 24 h, the alterations of the expression levels of phosphorylated and total Akt and Erk proteins were evaluated by western blotting. As shown in Fig. 5, the expression levels of phosphorylated form of each protein were prominently downregulated by ATRA and VD3, respectively, whereas almost no alteration was observed in their total form. Intriguingly, dasatinib per se did not affect the expression levels of phosphorylated form of each protein as compared to control group, and also showed no influence on ATRA- or VD3-triggered downregulation of respective protein expression level.

## Discussion

In this study, we first demonstrated that significant higher expression levels of total Lyn were observed in AML patients with favorable cytogenetics, higher MPO activity and FAB M2 subtype, which is also known as myeloblastic leukemia with maturation (Figs. 1, 2a–c; Table 1). Furthermore, a clear positive correlation was observed between the expression levels of Lyn and CD15 (Fig. 2d), suggesting for the first time that Lyn expression levels are closely associated with the differentiation status in AML blasts. Interestingly, a strong inverse correlation between the expression levels of Lyn and patients’ age was also observed, whereas no correlation was observed between its expression and the WBC numbers (Fig. 2e, f; Table 1). Therefore, our results suggest that the expression level of Lyn is closely linked to the differentiation status of AML blasts.

Our experimental data also showed that treatment with dasatinib alone clearly inhibited the expression of phosphorylated Lyn in HL-60 cells (Fig. 3a), consistent with a previous report showing that Lyn is the predominant activated SFK in HL-60 cells [12]. We further demonstrated that treatment with ATRA and VD3 resulted in differentiation induction in HL-60 cells as evidenced by the morphological changes and the increase in CD11b and/or CD11c expression levels (Fig. 3b–d). Although dasatinib per se did not clearly induced differentiation of HL-60 cells, the differentiation-inducing activity of ATRA was prominently enhanced by the addition of dasatinib (Fig. 3b–d). In agreement with our results, Congleton et al. and Kropf et al. have also reported similar phenomena, although the concentrations of dasatinib used in respective study were somewhat different [12, 14]. More important, we demonstrated that dasatinib also prominently enhanced the differentiation-inducing activity of VD3, which has been being studied as potential agent for differentiation therapy of AML. Since the concentrations of VD3 for in vitro and animal experiments far exceed those that can be tolerated by human beings [36, 37], it has not yet been translated to the clinic. To our knowledge, this is the first report regarding the combinatorial effect of VD3 and dasatinib in myeloid cell differentiation, and might provide fundamental insights for this combination therapy in patients with AML.

c-Myc is a transcription factor and its deregulation is common in leukemias and lymphomas in which overexpression of c-Myc occurs generally [19, 26]. Previous reports have demonstrated that repression of c-Myc is required for terminal differentiation of malignant hematopoietic cells including HL-60 induced by different stimulants such as ATRA and VD3 [23–26]. In agreement with these previous reports, our results demonstrated that downregulation of the expression of c-Myc protein was induced by either ATRA or VD3 alone (Fig. 4), thereby reconfirming a vital role of c-Myc in ATRA/VD3-induced HL-60 differentiation. Due to the critical role of c-Myc in differentiation and cell fate decision, it is tightly controlled through complex signaling pathways [19, 29]. It is noteworthy that two phosphorylation sites, threonine 58 and serine 62 exist in the N-terminus of c-Myc [19]. Threonine 58 is targeted by glycogen synthase kinase (GSK-3β) whose phosphorylation is known to be responsible for destabilization of c-Myc [28]. Moreover, inhibition of Akt signaling pathway is associated with the activation of GSK-3β, resulting in the degradation of c-Myc through the ubiquitin–proteasome pathway [19, 27]. On the other hand, serine 62 is a target of Erk whose phosphorylation is reported to stabilize c-Myc [19]. In the current study, along with the downregulation of c-Myc in the HL-60 cells treated with ATRA, VD3, each alone or in combination with dasatinib, downregulation of the phosphorylation of Akt and Erk was observed concomitantly (Fig. 5). Taking these previous results and our observations into account, we suggest that downregulation of c-Myc implicated with the repression of the activation of Akt and Erk may contribute to the induction of HL-60 differentiation by these reagents. More importantly, in comparison with the treatment with ATRA or VD3 alone, dasatinib significantly strengthened their ability to downregulate the c-Myc expression, although dasatinib per se did not affect the phosphorylation of Akt and Erk, even if when combined with ATRA or VD3 (Figs. 4, 5). It still remains uncertain and controversial with respect to the effects of dasatinib on the activation of Akt and Erk. A previous report has demonstrated that dasatinib downregulates the activation of Akt and Erk, contributing to its antitumor effects in nasopharyngeal carcinoma [38]. Furthermore, Chen et al. has demonstrated that dasatinib enhances cisplatin sensitivity in human esophageal squamous cell carcinoma cells via suppression of the activation of Akt [39]. On the other hand, similarly to our results, previous reports have demonstrated that dasatinib did not cause detectable differences in Erk and Akt in HL-60 cells [14] and human AML progenitor cells [40], respectively. It is worth noting that signal transducer and activator of transcription 3 (Stat3) has been suggested to be involved in dasatinib-triggered inhibition of c-Myc expression [39], suggesting a possibility of an implication of Stats3 for the downregulation of c-Myc expression. Overall, our results suggested that the mechanisms underlying downregulation of c-Myc by dasatinib clearly differ from that by ATRA/VD3. Collectively, we suggested that Lyn has a critical role in the negative regulation of the induction of HL-60 differentiation by either ATRA or VD3 through modulation of c-Myc expression, although the detailed mechanisms underlying the correlation are needed to clarify. Further investigation of the molecular details is ongoing.

## Conclusions

We demonstrated a clear positive correlation between the expression levels of Lyn and differentiation status of primary AML blasts. We further demonstrated that dasatinib not only enhanced the differentiation-inducing activity of ATRA but also that of VD3 in HL-60 cells, suggesting that Lyn was associated in the negative regulation of ATRA- or VD3-induced HL-60 cells differentiation. Our results also suggested that the enhanced differentiation-inducing activity probably was attributed to the downregulation of c-Myc implicated with the suppression of the activation of Akt and Erk in ATRA- or VD3-mediated HL-60 differentiation. These findings thus provide novel insights into a possible combinational therapeutic approach by targeting Lyn for AML patients, and offer new possibilities for the combination therapy with VD3 and dasatinib.

## Abbreviations

AML: acute myeloid leukemia
APL: acute promyelocytic leukemia
MPO: myeloperoxidase
ATRA: all-*trans* retinoic acid
VD3: dihydroxyvitamin D3
FAB: French-American-British
SFKs: Src family kinases
PE: phycoerythrin
FITC: fluorescein isothiocyanate
WBC: white blood cell
GSK-3β: glycogen synthase kinase
MFI: mean fluorescence intensity
NK: normal karyotype
MDS/AML: myelodysplastic syndrome overt acute myeloid leukemia
